# The U. S. Veterinary Medical Association

**Published:** 1885-10

**Authors:** 


### THE UNITED STATES VETERINARY MEDICAL ASSOCIATION.

The annual meeting of this Association was held at the American Veterinary College, on the 14th of September. The meeting was made notable by the general absence of the majority of its members. An election of officers took place, resulting in the election of Dr. McLean, Sr., of Brooklyn, as President; Dr. Cosgrove, of Mass., Vice-President; Dr. Robertson, of New York, Treasurer; and re-election of Dr. Minchener, of New York, as Secretary.

---

If one were asked, "what these meetings are held for?" it would be hard to answer, except to say for a sort of social coming together. For years they have worked by a decided poverty in papers or discussions of any value; hence, it is no wonder that members from a distance do not care to waste time and money by attending them.

It is an unquestionable necessity that *new life* must either be inspired in some way, or that the really active and earnest members of the profession branch off and form a new and more ultra-progressive association, where something can be and shall be done to demonstrate to the public that there is not only earnestness but knowledge, other than mere practical empiricism, among the men filling the ranks of the veterinary profession in the United States.

In other countries the men occupying the positions as teachers at the school make themselves known above and among their colleagues by the reading, discussion and publication of learned and progressive papers.

Those men occupying similar positions in this country have thus distinguished themselves in exactly the opposite way. With the exception of Drs. Liautard and McEachron, it is years since we have seen any publication from them, and from those at Harvard and Philadelphia we have yet to see the first indication that, notwithstanding the many advantages for material which their positions give them, they are in any way fitted for the positions they occupy, or that they possess any educational or intellectual abilities above the rank and file of the profession.

This is not right; when men assume such public and responsible positions, or honored by appointments which would tend to make the people think that they possess abilities superior to us common drones, they are in duty bound to show it, or else they are false to their obligations both to those that appoint them and the public. They do not deserve the honor that has been conferred upon them.

The profession has as much right to look upon such men as its teachers, by means of published writings, as the students have for their daily instruction.

The trouble is, that neither those making the appointments, nor those men that have been honored by receiving them, do not seem to have any idea of the true relation of the veterinary teacher in a school of medicine devoted to those subjects.

Such men should be obliged to give their whole time and entire energies to study, experiment and instruction.

They should be absolutely forbidden from taking any practice outside of the school hospital; or from visiting any patient except as a consulting physician, and at the request of an outside practitioner. If such men have not that professional enthusiasm which would enable them to devote their lives to their country's service for a simple living; if such schools have not the funds that should enable them to pay such remuneration, then the latter have neither right nor cause to exist, and the former should never accept such positions; for they have no professional right to take monetary advantages offered by the positions over less favored members of the profession.

